# 3-Methyl Thiophene-Modified Boron-Doped Diamond (BDD) Electrodes as Efficient Catalysts for Phenol Detection—A Case Study for the Detection of Gallic Acid in Three Specific Tea Types

**DOI:** 10.3390/foods13152447

**Published:** 2024-08-02

**Authors:** Dhielnawaaz Abrahams, Priscilla G. L. Baker

**Affiliations:** SensorLab Research Group, Chemistry Department, University of The Western Cape, P.O. Box X17, Cape Town 7535, South Africa; pbaker@myuwc.ac.za

**Keywords:** 3-methyl thiophene, boron-doped diamond, chemical sensor, electrochemistry, emerging contaminants, tea, total polyphenol content

## Abstract

Polymer modification has been established as a cost-effective, simple, in situ method for overcoming some of the inherent disadvantages of boron-doped diamond (BDD) electrodes, and its application has been extended to reliable, low-cost environmental monitoring solutions. The present review focuses on modifying BDD electrodes with semi-conductive polymers acting as redox mediators. This article reports on the development of a 3-methyl thiophene-modified boron-doped diamond (BDD/P3MT) sensor for the electrochemical determination of total phenolic compounds (TPCs) in tea samples, using gallic acid (GA) as a marker. GA is a significant polyphenol with various biological activities, making its quantification crucial. Thus, a simple, fast, and sensitive GA sensor was fabricated using the electroanalytical square wave voltammetry (SWV) technique. The sensor utilizes a semi-conductive polymer, 3-methyl thiophene, as a redox mediator to enhance BDD’s sensitivity and selectivity. Electrochemical synthesis was used for polymer deposition, allowing for greater purity and avoiding solubility problems. The BDD/P3MT sensor exhibits good electrochemical properties, including rapid charge transfer and a large electrochemical area, enabling GA detection with a limit of detection of 11 mg/L. The sensor’s response was correlated with TPCs measured by the Folin–Ciocalteu method. Square wave voltammetry (SWV) showed a good linear relationship between peak currents and GA concentrations in a wide linear range of 3–71 mg/L under optimal conditions. The BDD/P3MT sensor accurately measured TPCs in green tea, rooibos tea, and black tea samples, with green tea exhibiting the highest TPC levels. The results demonstrate the potential of the modified BDD electrode for the rapid and accurate detection of phenolic compounds in tea, with implications for quality control and antioxidant activity assessments. The prolific publications of the past decade have established BDD electrodes as robust BDD sensors for quantifying polyphenols. Fruits, vegetables, nuts, plant-derived beverages such as tea and wine, traditional Eastern remedies and various herbal nutritional supplements contain phenolic chemicals. The safety concerns of contaminated food intake are significant health concerns worldwide, as there exists a critical nexus between food safety, nutrition, and food security. It has been well established that green tea polyphenol consumption promotes positive health effects. Despite their potential benefits, consuming high amounts of these polyphenols has sparked debate due to concerns over potential negative consequences.

## 1. Introduction

Polyphenols are common secondary metabolites found in plants [1]. Recently, significant discussion and curiosity have been sparked about the possible health advantages of dietary plant polyphenols as antioxidants. The reaction of an antioxidant with a free radical results in the loss of an electron, the oxidization of the molecule, and the formation of a weak, non-toxic free radical that is stable and unable to further react [1,2]. Antioxidants have a wide range of functions in food matrices, including fat rancidity avoidance and reduced oxidative stress. Numerous research studies have stressed the significance of polyphenols, as their activity is linked to a sample’s (food’s) antioxidant capacity (AOC). Other studies have highlighted the importance of single molecules or groups of polyphenols in specific foods [2,3,4,5]. To avoid harmful side effects, extensive research must ascertain the optimal dosage for safe and beneficial use in various foods. Studies have shown the pro-oxidant and adverse effects of plant metabolites and polyphenol compounds, where high dosages of specific polyphenols have been linked to harmful side effects. The iron-chelating effects of epigallocatechin gallate (EGCG), curcumin, myricetin, ginsenosides, and ginkgetin are thought to be the underlying mechanism that allows polyphenols to prevent neurotoxicity. EGCG has also shown protein-binding properties that can inhibit the digestion of starch, lipids, and protein by interacting with and suppressing digestion enzymes in the gastrointestinal tract [6,7,8].

The possibility of consuming hazardous doses of polyphenols is particularly a concern for supplements. Several producers advocate for more than a 100 times greater intake than that which is typically linked with a standard diet. In specific research, antioxidant supplementation treatments have been linked to adverse outcomes such as higher mortality or strokes. In addition, concerns about variable effects on subpopulations and interactions with pharmaceuticals emerge when polyphenol consumption is encouraged at levels well above natural occurrence.

Food fortification can only be adequately informed with a comprehensive understanding of the protective and beneficial levels of polyphenol ingestion. Grape pomace extract contains a variety of polyphenols, which includes phenolic acids (gallic acid, its 3- and 4-β-glucopyranosides, trans-caftaric acid, and cis- and trans-coumaric acids), phenolic alcohol (2-hydroxy-5-(2-hydroxyethyl)phenyl-β-glucopyranoside) and flavan-3-ols (catechin, epicatechin, and procyanidin B1). Grape extract, with a polyphenol content of 100 or 500 μg/mL, shows pro-oxidant and cell toxicity effects that result in anti-inflammatory properties in vitro [7]. Toxicological studies on green tea extracts, which are rich in catechin phenolic profiles, outlined the adverse effects of hepatotoxicity associated with (–)-epigallocatechin-3-gallate consumption at levels of 140 mg to 1.000 mg/day [9]. These examples highlight the need to reassess food application regulations to ensure acceptable levels during long-term product use and guarantee consumer safety.

## 2. Phenol Compounds as Priority Pollutants

Phenols are known to be toxic and have severe short- and long-term effects on humans and animals. Therefore, these compounds are undesirable contaminants of the environment in high concentrations. The United States Environmental Protection Agency (US EPA) and the European Union (EU) classify these compounds as pollutants of priority in the field of water policy by the European Water Framework Directive [10].

Many phenols are naturally occurring, while others are synthesized. In nature, they are constituents of coal tar and creosote oil, human/animal wastes, and decomposing organic material. They are found in many non-food and food sources, which are significant primary sources of phenols, making their way into the environment by disposing of wastewater (agricultural, industrial, municipal wastewater, and domestic activities) into the environment and atmosphere. They may also be present due to the degradation or decomposition of the naturally occurring organic matter that is in porewater, by discarding industrial and domestic wastewater into water sources, and finally through the runoffs from cultivated agricultural lands [11,12,13,14,15]. Wastewater protocols have been established to monitor and remove these chemical pollutants from primary water sources to minimize the effects of these chemicals on human and aquatic life. However, these methods are costly and time-consuming for priority contaminants, and their effectiveness for phenols and phenol-related compounds is often limited and inconsistent [10,12,15,16,17].

Phenols possess a wide range of chemical and physical properties, and their successful removal varies greatly depending on their particular properties. Due to wastewater’s significantly contaminated nature, wastewater treatment is a far more involved procedure than water treatment and it must be thoroughly treated before safely integrating it into the environment [15].

The recovery of phenolic compounds from aquatic systems is a prerequisite to safeguard the life of humans and marine organisms from contamination by these toxic chemicals. Implementation of appropriate technologies for the effective removal of these compounds includes centrifugal sedimentation, extraction, evaporation, polymerisation, ion exchange, oxidation with ozone, electro-Fenton process, solvent extraction, photocatalytic degradation, chlorination, stripping and oxidation, and aeration [16,18].

The most widely repeated method for phenol recovery is high-performance liquid chromatography (HPLC), with rescues in the range of 85.5% to 105.2% of phenol recovery [10]. The EPA prescribes a concentration of 1 mg/L in drinking water and has designated phenol as the 11th of the 126 priority pollutants. The phenol and polyphenol derivatives can have even more harmful effects than the original compounds. This transformation occurs due to the interaction of the phenols with physical, chemical, biological, or microbial factors in the water of interest [10].

Among aqueous industrial effluents, those containing organic pollutants are seen as problematic due to the high activation energy barrier needed to overcome chemical oxidation. The worldwide production of phenolic compounds stands at over 300.000 tonnes per year. A total of 60% of the global phenolic load is introduced into sewage and released into the environment, where 85% is in the form of potentially oestrogenic degradation products [10].

Phenolic compounds can easily find their way into humans’ gastrointestinal tracts and penetrate the skin [19]. In vivo, they can transform into various reactive intermediate forms, particularly quinone moieties, which form covalent bonds with proteins, resulting in cancerous effects [19]. Phenol and its methyl derivatives adduct strongly to adsorb onto solid matrices and aquatic life at very low concentrations [19].

## 3. Health Risk of Ingesting Polyphenols

Most research into polyphenols is driven by their ability to counteract infection, disease, or toxic drugs, as polyphenols have long been associated with aiding good health [20]. Toxicity studies are limited and primarily based on in vitro or animal studies. The toxic actions of polyphenols have been identified as pro-oxidant, oestrogenic, immunosuppressant, carcinogenic and genotoxic for specific phenolic compounds. These studies established that frequently consumed dietary polyphenols could exert harmful effects at elevated concentrations, which may be exacerbated by interactions with other pharmacological agents [20,21,22,23].

Low concentrations (1.0 × 10^−5^–2.0 × 10^−5^ mol/dm³) of the anthocyanins pelargonidin, cyanidin, and delphinidin have been linked to oestrogenic and antioestrogenic actions through oestrogen induction of cell proliferation. However, they decrease oestradiol-induced cell proliferation at elevated concentrations and in combination with endogenous oestrogen–oestradiol [20].

Kaempferol and resveratrol have been observed to demonstrate oestrogenic activity at concentrations of 1.0 × 10^−6^–1.0 × 10^−5^ mol/dm³ and 10^−8^–10^−4^ mol/dm³, respectively. In addition, both phenols have been reported to increase the growth of breast cancer cells at these low concentrations. However, at higher concentrations (3.5 × 10^−5^ and 7.0 × 10^−5^ mol/dm³), kaempferol reportedly reduces the number of viable cells significantly [20].

Polyphenols, generally regarded as antioxidants, can have a pro-oxidant nature. They perform this function by initiating an auto-oxidation process and behaving like a pro-oxidant. This pro-oxidant effect occurs at high pH, temperature, the presence of iron, and a high concentration of the specific phenolic compound [20].

Epigallocatechin gallate (EGCG), found in green tea leaves, is the ester of epigallocatechin and gallic acid that undergoes oxidative polymerisation in vitro and causes the production of toxic hydrogen peroxide (H_2_O_2_). In liver cells and tissues treated with a high level of EGCG (200 μmol/L), cell viability drastically decreased due to the increased reactive oxygen species (ROS) production and reduction of glutathione [20,24] Polyphenols, like pro anthocyanidins, which are typically found in green tea and at lower concentrations in black tea, are considered antinutritional compounds that may inhibit iron absorption. Rooibos tea is free of anthocyanidins and does not significantly affect iron absorption. Rooibos tea also does not contain epigallocatechin gallate (EGCG) [20,25,26].

## 4. BDD as a Transducer for Phenol Detection

Traditional analytical methods are plagued by inherent impedance to efficient analysis, such as complex pre-treatment, complicated and expensive instrumentation, time-intensiveness, and the need for professional operators. Electrochemical methods simplify these challenges and are relatively cost-effective in instrumentation. They deliver a fast response and save time for bulk analysis.

The superior characteristics of boron-doped diamond (BDD) electrodes and the performance quality of BDD sensors have caused electrochemical sensors based on BDD electrodes to spark widespread attention. As with carbon-based electrodes, electrochemical reactions performed at the interface between electrolyte solutions and the electrode surface may alter the BDD electrode’s surface structure and properties [27].

Compact reviews, research articles, and books on boron-doped diamond electrode electrochemistry have been published in the last ten years, including diamond nanostructures and particles [28,29,30,31,32,33,34,35,36,37,38,39]. Moreover, outputs of the twenty-five-year history of BDD-related research (10,000 publications according to the ScienceDirect database) can be traced in several reviews devoted to particular aspects, such as surface modifications [29,40,41,42,43,44,45,46,47,48], electrosynthesis and anodic wastewater treatment, electroanalytical applications, including applications in biosensors [49,50,51,52,53,54,55], and liquid-flow techniques employing BDD electrodes for electrochemical detection [36,56,57,58,59,60,61,62].

**Figure 1:**
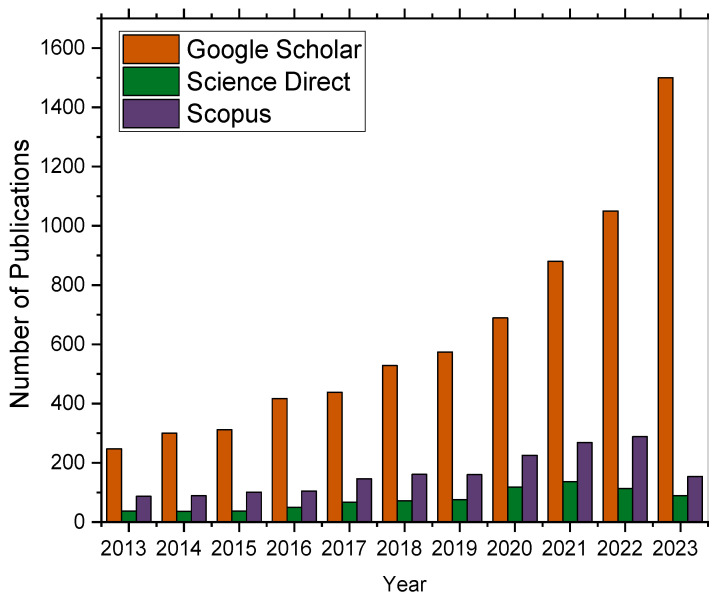
Number of publications in three scientific databases (Google Scholar, Scopus, and Science Direct) issued from 2013 to March 2023 related to keyword searches for “boron-doped diamond electrode, polymer, and phenol detection”.

The histogram in Figure 1 displays notable highlights from the past decade of research, including the application of BDD electrodes to extracts obtained from natural products, BDD electrodes in coupled detection methods, nano-structuring of BDD electrodes for enhanced performance, and numerous surface modification strategies.

Wedelolactone is a polyphenol isolated from *Wedelia chinensis* and *Eclipta alba* and was evaluated as a potential anti-HIV herbal drug. Under diffusion-controlled electrochemical behaviour, cyclic voltammetry at the boron-doped diamond surface indicated the oxidation of wedelolactone with two oxidation peaks (P1 and P2) with Ep_1_ = 0.4 V and Ep_2_ = 1.00 V. The analytical response of wedelolactone at the BDD electrode was linear in the concentration range of 50–700 ng/mL, with a limit of detection (LOD) and limit of quantitation (LOQ) of 43.87 and 132.93 ng/mL, respectively, in real plant samples [63].

The paper presents a voltammetric method for determining wedelolactone, an anti-HIV herbal drug, using a boron-doped diamond electrode, offering a sensitive and precise tool for quality control, pharmaceutical analysis, research, herbal medicine standardization, and anti-HIV drug development [63].

Many studies have also been directed towards understanding the electrochemical degradation pathway of phenols at BDD by combining voltammetric studies with bulk electrolysis, UV spectroscopy, HPLC analysis, chemical oxygen demand (COD), and total organic carbon (TOC) measurements, often using gallic acid as a model compound [64,65,66]. Both direct and mediated electrochemical processes were identified in the oxidation of gallic acid, and the degradation of gallic acid was characterized by pseudo-first-order kinetics. Aliphatic acids were the primary intermediates formed during the electrolysis and were finally mineralized to CO_2_ and water [66]. Hayes et al., 2019 presents a chromatography method with electrochemical detection using a BDD electrode that is applicable in pharmaceutical analysis, environmental monitoring, food safety testing, and biomedical research, offering high sensitivity, stability, and resistance to fouling for the accurate and precise detection and quantification of specific compounds in complex samples [66]. Combining the discriminatory power of the electrochemical degradation of phenols at BDD electrodes with the high-performance liquid chromatography (HPLC) method delivered good linearity for up to nine phenols, with high precision and nanomolar detection in Islay, Irish, Scotch, and Highland whiskey samples [66,67]. In addition, HPLC with spectrophotometric detection delivered comparable results to HPLC with amperometric detection, depending on the analyte under investigation [42].

Cyclic voltammetry and amperometric detection coupled with flow injection analysis (FIA-EC) were used to study the electro-oxidation reaction of chlorinated phenols at microcrystalline and nanocrystalline diamond thin-film electrodes, yielding low mass limits of quantification (100–1000 pg) for all the single chlorinated phenols. Furthermore, both diamond electrodes outperformed glassy carbon, which exhibited short-lived reduced consequences of fouling by reaction products and potential-dependent changes in the electrode’s physicochemical properties [66]. Researchers developed a highly efficient electrode for electrochemically oxidizing chlorinated phenols, which are toxic pollutants, using microcrystalline and nanocrystalline boron-doped diamond electrodes. This innovation has applications in water treatment, environmental remediation, industrial processes, sensor development, and pollution control. The electrode’s high electrochemical activity, stability, and durability make it a valuable tool for removing chlorinated phenols from the environment [64,65,66,67,68].

Boron-doped diamond nanowire (BDDNW) electrodes were produced by the metal-assisted chemical etching of Si and electrostatic self-assembly nanodiamond seeding to provide a large surface area during electrochemical oxidation. As a result, the effective surface area calculated from cyclic voltammetry was enhanced several times greater than that of a standard planar BDD electrode, and a much lower energy consumption was observed in the phenol oxidation process [68]. The development of BDDNW electrodes has revolutionized the electrochemical oxidation of phenol, a harmful pollutant. By enhancing the oxidation process, this innovative electrode offers a highly efficient solution for removing phenol from wastewater and industrial effluents, thereby contributing to effective water treatment and environmental remediation. Furthermore, its exceptional stability and durability make it an attractive option for various industrial applications, sensor development, and energy storage solutions, paving the way for a more sustainable and environmentally friendly future [68].

Molecularly imprinted polymers, also termed synthetic antibodies, are polymers that have been produced to bind to a molecule of interest selectively. This approach has been exploited in the design of catechol-imprinted chitosan films electrodeposited onto BDD electrodes. The performance of the sensor showed excellent reproducibility (RSD = 4.1%) and repeatability (RSD = 7.0%) for catechol detection in the range of 0 to 80 μM, with a detection limit of 6.9 × 10^−7^ M and high selectivity to catechol recognition when applied to red wine. The molecularly imprinted polymer sensor on a boron-doped diamond electrode was designed for the highly sensitive and selective detection of catechol in environmental, industrial, food, medical, and pharmaceutical applications [69,70,71,72].

## 5. 3-Methyl Thiophene-Modified Boron-Doped Diamond (BDD) Transducer for Phenol Detection

BDD electrodes and BDD anodic oxidation have demonstrated good electrochemical performance. They have strong oxidation stability, a very low-capacity background, high current efficiency, a large potential window, weak electrode fouling, strong chemical and physical stability, and a long service life [29,56,73,74]. These have resulted in it becoming desirable and widely used in applications such as water treatment, biosensors, and electrochemical preparation. However, the BDD anode system is still not applied extensively [29,54,56,74,75].

Despite these unusual properties, the evolution of electrode fouling is found to be inevitable, even on the BDD electrode.

It has been seen that unmodified working electrodes or bare solid working electrodes utilized in the electrochemical oxidation of polyphenol compounds create a thin polymeric film on the electrode surface. This formation of the film causes the electrode to deactivate, resulting in the phenomenon known as “electrode fouling”. The process is based on a polymerisation reaction that occurs as phenol or semi-quinone radicals are formed upon the oxidation of phenolic and polyphenols compounds. This radical kickstarts the polymerisation reaction that in turn creates the polymeric layer. Direct oxidation of polyphenol compound will be inhibited as the electrode surface is then fouled. The electrode performance will be greatly affected by the generation or occurrence of a polymeric film on an electrode surface, which will have a detrimental effect during the analysis of phenols [42,76].

Many types of research have reported that using electrodes modified by intrinsically conducting polymers (ICPs) can prevent electrode fouling and consequently improve reliability and repeatability [77].

ICPs based on nanostructured materials have been recently developed and have led to a major increase in the quality of performance of sensors and biosensors, including the induction of direct load transfer processes between the biological product and the transducer. In the literature, polythiophene alteration is seen to avoid severe electrode fouling, which typically affects unmodified or bare electrode surfaces. Additionally, it also greatly increases the sensitivity of the amperometric sensor and is also seen to capture well-repeated voltammetry signals for phenol oxidation [77]. Furthermore, the ICP is considered desirable as a polythiophene, as it is characteristically easy to oxidize, has high chemical stability, provides homogeneous fabrication, and is soluble in many media (such as aqueous, acetonitrile, and propylene carbonate), therein fulfilling most of the requirements for a sensor [78].

Much of the focus has been on poly(3-methyl thiophene) (P3MT), a polythiophene derivative, due to its high conductivity, flexibility, high tensile strength, and versatility. P3MT has been investigated and applied in microelectronics, optoelectronics, and sensors, as it can be prepared by either chemical or electrochemical oxidation of the monomer with or without a template with varied nanostructures [79].

P3MT is correlated with the simplest and smallest alkyl group, methyl, with the formula CH3^−^. It produces increased conductivity over the unsubsidized monomer and stays strongly conductive by the incorporation of longer alkyl groups. Methylthiophene films are known to be very light. This is advantageous as a decay of conductivity is associated with longer alkyl chains. This weakening of conductivity is due to the presence of a larger proportion of the polymer molecule being an insulating hydrocarbon chain. Thiophene films with longer hydrocarbon chains have structures that are less regular and more porous [79].

In this study, we propose the development and testing of a chemical sensor on a BDD platform that can measure phenol levels in three different types of tea samples (namely rooibos tea, black tea, and green tea), as this will greatly facilitate the measurement and control of the total phenol content. The sensing principle is based on the determination of the sensing response to the phenol voltammetric method. Square wave voltammetry (SWV) is employed to investigate the activity of the BDD electrode for phenol determination in solution under varying experimental conditions, and to study the sensor’s oxidation response to phenol. The oxidation response of the sensor to the phenol has been studied.

To combat fouling and improve the superior electrochemical sensor, we have suggested the possibility of using BDD electrodes chemically modified with a conductive P3MT film. The electroactive properties of P3MT are provided with a high degree of cross-selectivity and with reasonable reproducibility [56,80].

Notably, methylation, glucoside acidification, and sulphated products are the main forms of polyphenols in vivo.

## 6. Case Study: Polymer-Modified BDD Sensors for the Detection of Phenols in South African Tea Samples

Gallic acid (3,4,5-trihydroxy benzoic acid) is the most popular hydroxybenzoic acid. It is among the most representative phenolic-type compounds present in numerous dietary substances and food wastewaters (agricultural (tea) effluent, olive oil factories, boiling cork process, and other industrial synthetic effluents) [81,82,83,84]. Gallic acid (GA) is widely known for its excellent antioxidant and antiradical activity in multiple biological and pharmaceutical applications owing to its structure. It is favoured for its antibacterial, anti-mutagenic, antiviral, anti-inflammatory, antihistaminic, antitumor, anti-cancerous and anti-diabetic properties, as well as protection against cardiovascular diseases [85]. GA is broadly utilised as a standard for the assurance of total polyphenolic substance in several analytes by the Folin–Ciocalteu test [82,83]. Different strategies have been utilized for the self-assurance of GA, such as high-performance liquid chromatography (HPLC), stream infusion examination, a switch stream spectrophotometry photoelectrochemical strategy, and electrochemical strategies [85,86,87,88]. The quantification of polyphenols at carbon nanomaterials, metal oxides, and redox polymers by electrochemical means is expressed as gallic acid equivalents (GAEs) [81,89,90,91].

Black and green tea are among the most common teas globally [92,93,94]. Annual tea production output is predicted to be 3.92 million tonnes, with black tea accounting for 60% of the total production and green tea accounting for 30%. It is expected that green tea will increase at a faster rate than black tea and other minor tea types [95]. The chemical composition of tea changes with temperature, season, variety, climate, horticultural practices, and leaf age, implying that tea grown in various regions across the world may have varying quantities of bioactive chemicals [82,83]

Tea polyphenols, often called catechins, comprise up to 30–42% of the dry weight of the solid particles in brewed green tea. Catechins are differentiated by the substitution of di- or tri-hydroxyl groups in the B ring and the substitution of meta-5,7-dihydroxy in the A ring. The most prevalent catechin in green tea is (−)-epigallocatechin gallate (EGCG), which accounts for 50–80% of the total amount of catechin in tea. The presence of the three other significant catechins in tea is of importance as well; they are (−)-epigallocatechin (EGC), (−)-epicatechin gallate (ECG), and (−)-epicatechin (EC). Smaller amounts of catechin, gallocatechin, epigallocatechin digallates, epicatechin digallate, 3-O-methyl (−)-epicatechin, (−)-epicatechin gallate, catechin gallate, and gallocatechin gallate are also found.

In the production of black tea, about 75% of catechins in the tea leaves undergo enzymatic transformation consisting of oxidation and partial polymerisation. In the manufacture of black tea, the tea leaves are crushed to allow the polyphenol oxidase to catalyse the oxidation, leading to the polymerisation of catechins. The resultant black tea composition is determined by the technological process of its manufacturing method. Therefore, stating a definitive composition for black tea beverages is difficult, as it varies with different preparations. The concentrations of catechins (10–12%), theaflavins (3–6%), thearubigins (12–18%), flavonols (6–8%), phenolic acids (10–12%), amino acids (13–15%), methylxanthines (8–11%), carbohydrates (15%), proteins (1%), mineral matter (10%), and volatiles have been reported [93].

Rooibos tea, indigenous to South Africa, has gained much popularity in the herbal tea market. It is made from the leaves and delicate stems of *Aspalathus linearis*, which includes aspalathin orientin and (+)-catechin.

The Folin–Ciocalteu technique is a well-established method for evaluating the total polyphenol contents of dietary samples, with findings represented as gallic acid equivalents (GAEs) [81,83]. Numerous research papers have been published on the total polyphenol contents of traditional/fermented and green/unfermented rooibos. Bramati et al. (2003) reported a level of 68.4 mg GAEs/g for green rooibos and 35.2 mg GAEs/g for traditional rooibos prepared as a 1% (*w*/*v*) aqueous extract with a steeping time of 10 min [96].

### 6.1. Material and Methods

In this particular case study, a selection of tea was obtained commercially from supermarkets in Cape Town, South Africa, as a representation of the main types of tea consumed in South Africa. This included black tea (Joko^®^), green tea (Woolworths Food^®^), and red rooibos tea (Joko^®^), which South Africans widely consume. The content of tea in any given teabag was weighed, dissolved in 100 mL of recently boiled water, and left to steep for 10 min. The tea extract was then filtered and left to cool at room temperature. Tea extracts were diluted with buffer (0.1 M phosphate buffer solution, pH 6.6) at ratios of 3:1, 3:2, 3:3, and 3:6 to find the optimum balance between electrolyte conductivity and sample responsiveness. In screening the samples for redox activity using cyclic voltammetry, the most pronounced electrochemical signals were observed for the samples prepared as a 3:2 tea to buffer ratio, respectively. This ratio was used for all tea samples; ten tea bags were screened per tea type. From the initial sample size of 30 tea bags, only those samples that registered an electrochemical profile in the potential window (−200 and +1500 mV at a scan rate of 50 mV/s (versus Ag/AgCl)) were retained for the quantitative assessment of phenols. Rooibos tea produced 3/10 samples for the phenol content analysis, whereas green tea produced 10/10 and black tea produced 6/10.

Commercial boron-doped diamond disk electrodes (3.0 mm diameter) were used as working electrodes (Windsor Scientific Limited, Reading, Berkshire, UK). Alumina micropolish and polishing pads (Buehler, Lake Bluff, IL, USA) were used for electrode polishing. All chemicals used were of analytical grade. Stock 0.5 M 3-methyl thiophene (Aldrich, St. Louis, MO, USA, 99%) solutions were prepared with 0.1 M lithium perchlorate (Aldrich, St. Louis, MO, USA) in pure acetonitrile (HPLC grade, Aldrich, St. Louis, MO, USA, 99%). All solutions were prepared using MilliQ UHQ water. The polyphenol stock solutions with a 0.1 mM concentration were prepared fresh before each experiment.

The BDD disk electrodes were pre-treated via an activation step that required repeatedly cycling the electrode via cyclic voltammetry in 1 M acetic acid from −200 mV and +1500 mV at 50 mV/s versus Ag/AgCl until a stable signal was detected. This activation resulted in introducing hydroxyl groups (OH^−^) at the surface of the BDD electrode, making them more hydrophilic. The activated electrode was then further modified by the electrodeposition of a thin film of poly-3-methyl thiophene (P3MT) from a 0.5 M monomer solution made of a 0.1 M lithium perchloride electrolyte solution. Between measurements, the electrode was subjected to electrochemical cleaning with 0.5 M H_2_SO_4_ for ten cycles at a scan rate of 100 mV/s to remove adsorbed species from its surface and render the commercial BDD electrode ready for repeated use.

For analytical purposes, cyclic voltammetry measurements were performed in a classical three-cell electrode cell, with the poly-3-methyl thiophene-modified BDD electrode (BDD/P3MT) connected as a working electrode, an Ag/AgCl reference electrode, and a Pt wire as a counter electrode (100 mm length, 0.4 mm diameter). The prepared electrodes were treated by repeated cycling (−200 mV to +1500 mV, scan rate of 100 mV/s) in pH 6.6 PBS until a stable background was obtained (10 cycles).

All electrochemical evaluations were performed at ambient temperature using a BASS electrochemical workstation and a conventional three-electrode cell.

#### 6.1.1. Characterization of the P3MT Thin Film by Cyclic Voltammetry

**Figure 2:**
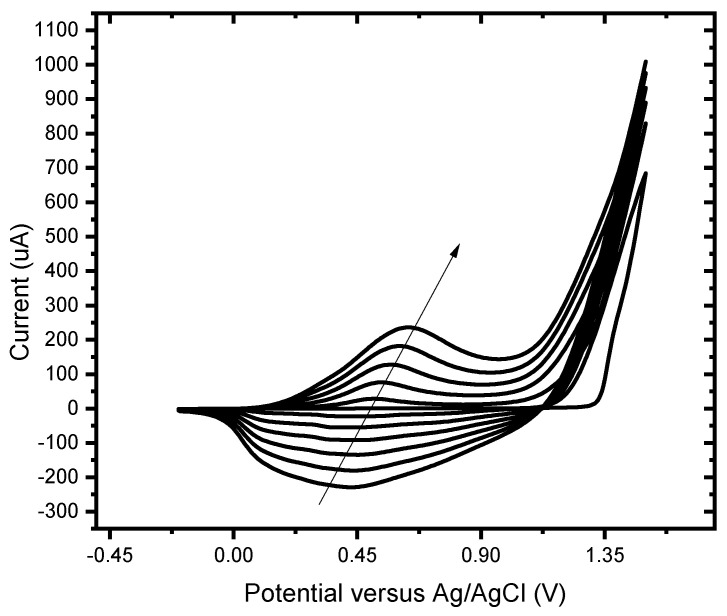
Cyclic voltammogram of the electro-polymerisation of poly-3-methylthiophene from 0.5 M 3-methyl thiophene at BDD in 0.1 M LiCO_4_ in acetonitrile at 50 mV/s for 5 scans.

Polymer formation can be followed by cyclic voltammetry (Figure 2). As the applied potential surpasses 100 mV, an increase in the anodic current is observed, followed by a current plateau that maintains a positive current slope, and an anodic hysteresis loop is displayed, characteristic of electrochemical reactions involving nucleation and growth of the new phase. Subsequently, potential cycling produces changes in the anodic and cathodic currents, and the electrode is covered with a uniform insoluble and adherent deposit. The electrochemical synthesis of the P3MT polymer demonstrated that the electrochemical nature is strongly influenced by the mechanism of oxidation. The two redox couples observed in the CV voltammogram obtained for the electrochemical synthesis show that the monomer undergoes oxidation to form a radical cation, which then further oxidizes to form a dication. These two species combine simultaneously to form the polymer thin film.

**Figure 3:**
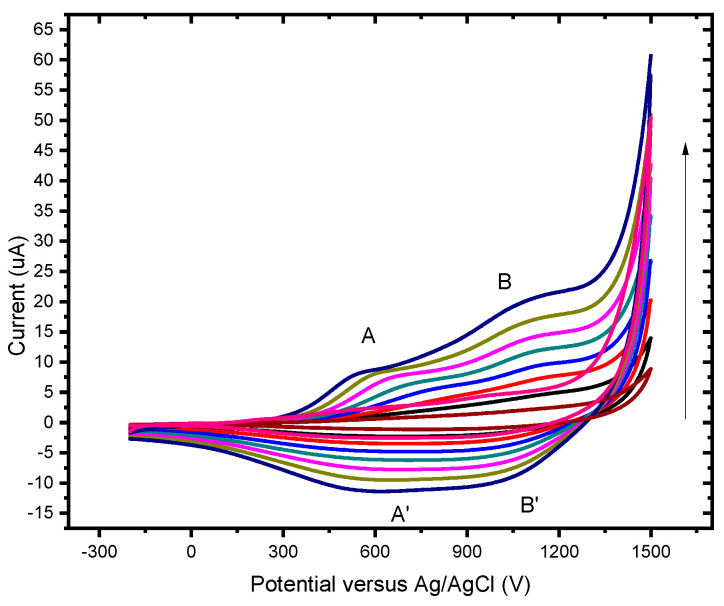
Cyclic voltammograms of poly (3-methyl thiophene) at BDD in 0.1 M LiCO_4_ in acetonitrile at different scan rates: from inside to outside 10, 20, 30, 40, 50, 60, 70, 80, 90, and 100 mV/s.

**Figure 4:**
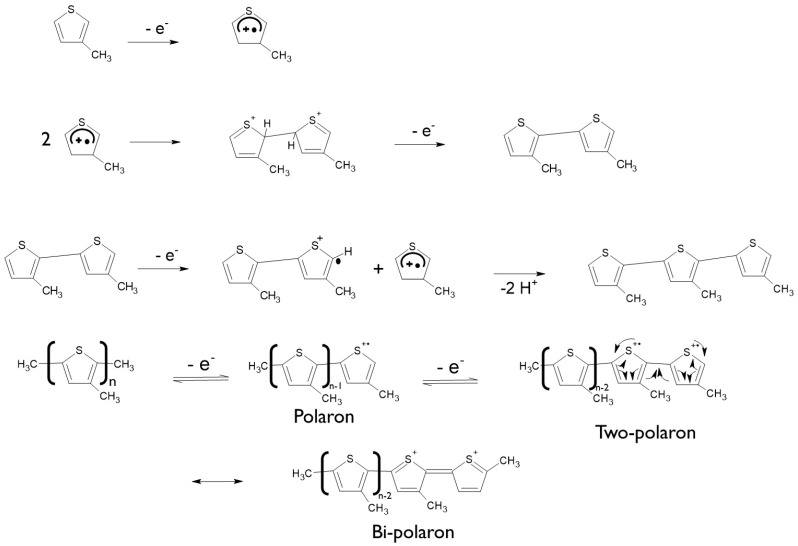
The electrochemical synthesis pathway of 3-methyl-polythiophene via cyclic voltammetry.

The 3-methyl thiophene monomer unit has a CH_3_ (methyl) group located at the beta position of the thiophene ring. Although only a small alkyl chain is introduced, many properties of the polymer change, including generating a lower oxidization potential (+600 mV). The polymer layer was redox-active (Figure 3 exhibits two reversible and clearly defined pairs of redox peaks). Redox couples A/A″ and B/B″ were attributed to the cation radical coupling mechanism. The oxidation of the monomer at the BDD anode surface produces charged species precursors. These charged species require the transfer of two electrons per molecule for electro-polymerisation. In addition, the radical cations produce a dihydro-dimer cation, leading to a dimer after the loss of two protons and aromatization. This aromatization constitutes the driving force for the polymer film formation (Figure 4). An examination of 3-methyl thiophene with increasing growth cycles demonstrates the signal increases as more material is deposited and the potential gap is extended between the redox responses. Over three or more growth cycles, the appearance of a pre-peak is visible; however, upon slower scan rates with a film generated through a single potential cycle, the pre-peak is also evident. The pre-peak seen through the poly(3-methyl thiophene) voltammogram series is reported in the literature and has been attributed to the presence of different conjugation lengths and the presence of two conformers. Additionally, relaxation effects linked to reversible polymer swelling, resistivity changes, and electronic structures, relating to bipolaron, polaron plus bipolaron, and pi-dimers, have all been postulated in the attempt to identify and explain the polymer behaviour.

**Figure 5:**
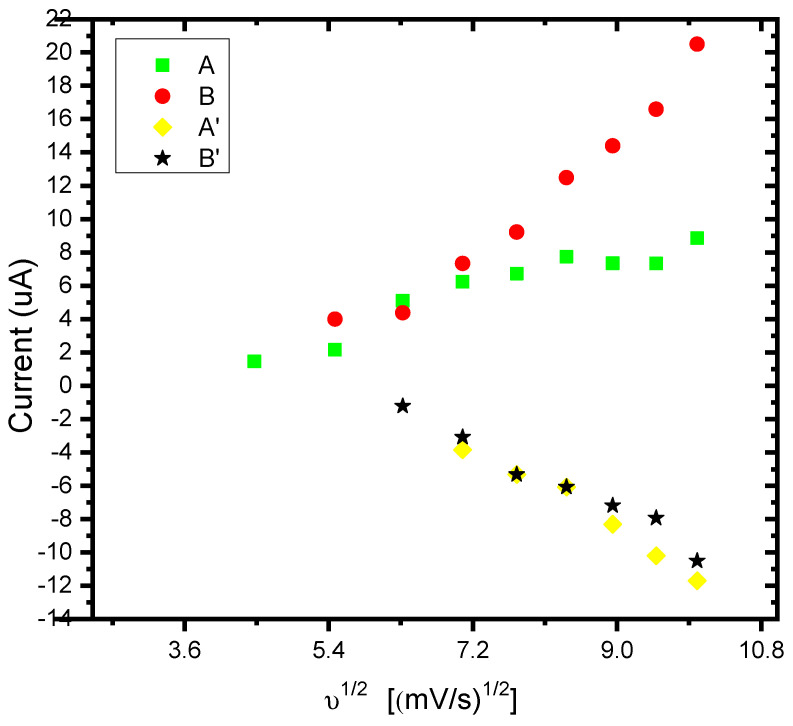
The linear variation in the anodic and cathodic peak currents as a function of the square root of scan rates for poly (3-methyl thiophene) in 0.1 M LiCO_4_ in acetonitrile.

The linear dependence of the peak currents of anodic peak A on the scan rate indicated that we have a thin film of the surface-bound conducting electroactive polymer undergoing a rapid, reversible electron transfer reaction [97]. Figure 5 illustrates the dependence plot of the cathodic peak current (I_p,c_) as a function of the square root (υ^1/2^) of the potential scan rate (correlation coefficient, R^2^ = 0.97 and slope 2.5) for the P3MT film, related to the peak labelled B. This confirms the diffusion-controlled cathodic peak current arising from the electron propagation through the polymer chain. The Randel–Sevćik equation was used to determine the diffusion coefficient of the electrons (D_e_) within the polymer. The slope of the straight line derived from the I_p,c_ versus υ^1/2^ plot (Figure 4) may be used to calculate D_e_. The diffusion coefficient (De) was calculated to be 3.6 × 10^−10^ cm^2^/s for the polymer film [77,97,98,99,100]. The increasing amplitude of the redox peaks with repeated potential scans indicated that the polymer deposited at the BDD surface was conducting.

#### 6.1.2. Morphology

##### Scanning Electron Microscopy (SEM) Imaging of the Polymer Layer

**Figure 6:**
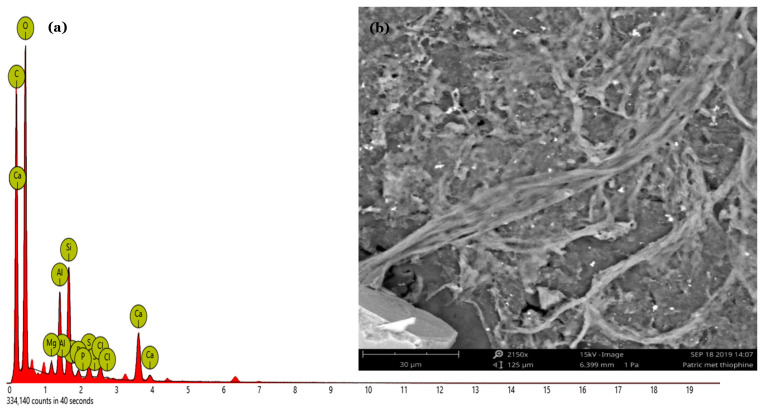
(**a**) SEM-EDX obtained for the P3MT thin film. (**b**) SEM micrograph of the polymer film at high magnification (32 µm).

The polymer-modified electrode morphology was rough, with a compact and homogeneous structure (Figure 6). Irregular round granules of various sizes (3 μm, 5 μm, and 30 μm) were observed for the P3MT films.

SEM imaging was conducted using a Phenom Pharos G2 Desktop FEG-SEM and a FEG source with 1–20 kV acceleration voltage range, a high resolution (fegsem) field emission gun scanning electron microscope. SEM coupled to elemental analysis was utilized to examine the polymer layer using backscattered electrons, which generate pictures with contrast that carry information about atomic number differences. Secondary electrons generate topographic information about the material. Combining the SEM instrument with an EDX detector allows X-rays to be used as a signal to generate chemical information on the investigated material. SEM-EDX is a secondary electron backscatter capture technology used to determine a polymer’s chemical composition. It is also used to identify elements and offer quantitative compositional information. The instrument was rapid, easy to use, and non-destructive and was used to validate the presence of sulphur arising from polymer film formation [64,65].

Atomic force microscopy (AFM), also known as scanning force microscopy (SFM), has been used since the initial stages of imaging nanoscale materials. It produces topographic information of analysed samples on a nanometre scale resolution for measurement and other properties. A very high-resolution scanning probe, called a cantilever, is employed, producing a resolution on the order of fractions of a nanometre. The force between the cantilever and the sample is measured, and this force is then used to generate a topographical image of the samples. The Nanosurf EasyScan 2 is an atomic force microscope system used to attain topographical and roughness information on the unmodified and modified thin films.

**Figure 7:**
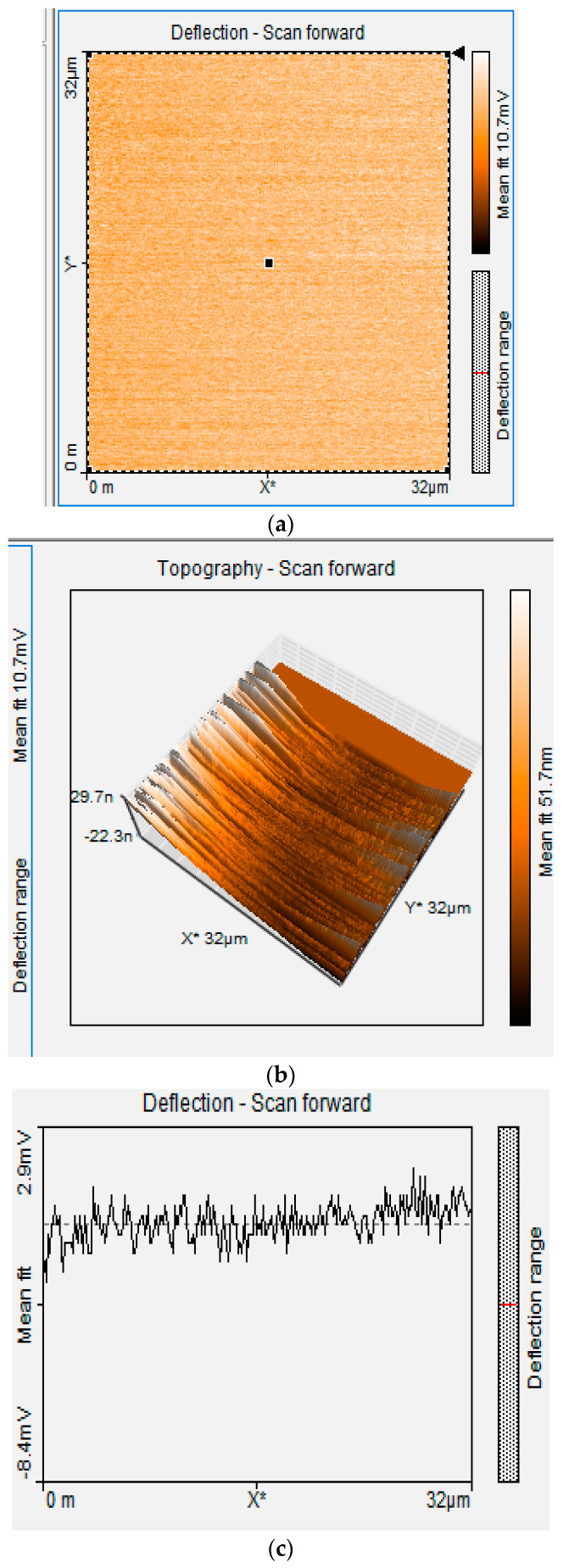
AFM image of the unmodified BDD at 32 × 32 μm at 500 mV. (**a**) Topographic 2D scan, (**b**) topographic 3D scan, and (**c**) deflection scan.

**Figure 8:**
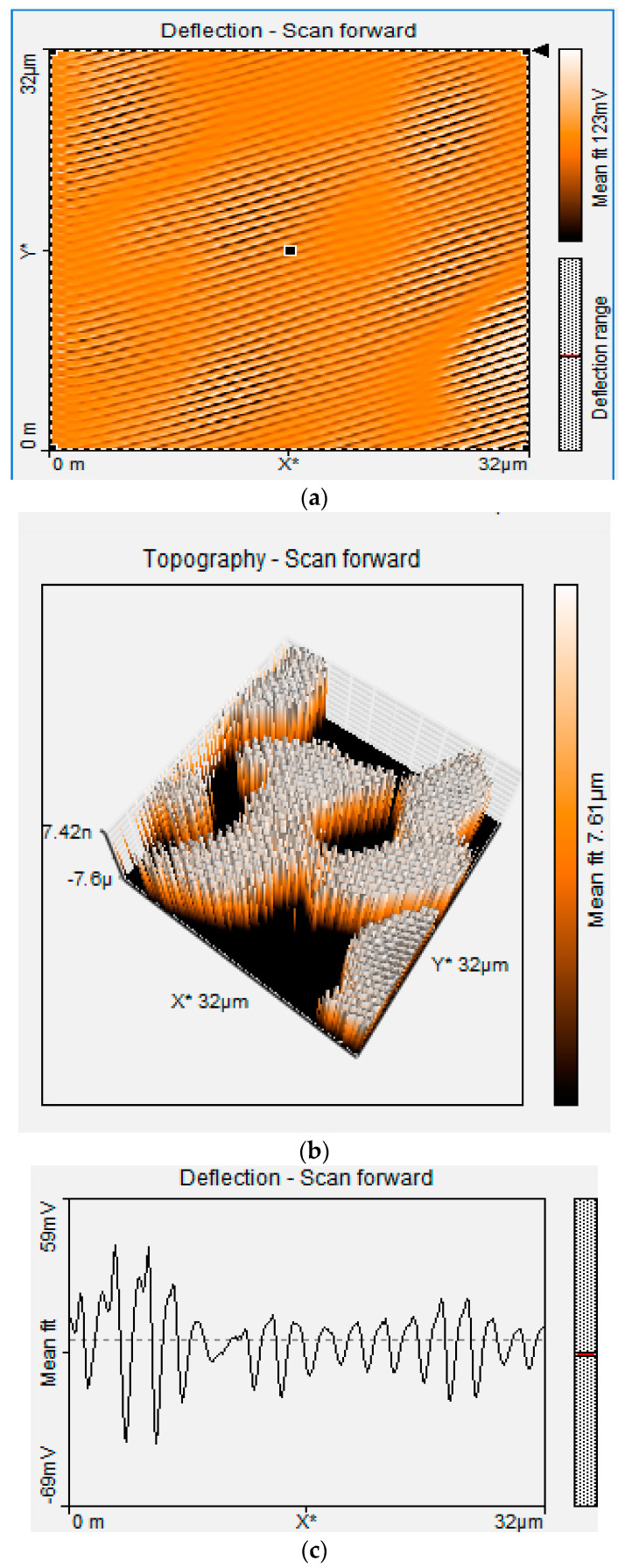
AFM image of BDD/P3MT at 32 × 32 μm at 500 mV (**a**) Topographic 2D scan, (**b**) topographic 3D scan, and (**c**) deflection scan.

The AFM results shown in Figure 7 and Figure 8, indicates morphological changes in the boron-doped diamond surface, as indicated by a change in roughness at the nano- and microscale levels. The AFM image shows a smooth surface for the unmodified BDD. At the same time, textural changes are observed for P3MT, which is evident in the granular-shaped structures with some irregular forms observed in the topological image under our experimental conditions [65].

Table 1 presents the results of roughness assessments of unmodified BDD and P3MT/BDD film surfaces using Atomic Force Microscopy (AFM), providing information on surface areas and corresponding roughness values. A smooth surface was observed for unmodified BDD in all image sizes (32 × 32 μm and 8 × 8 μm). The voltage applied was 500 mV and 1000 mV. Generally, the deflection scan’s low values correspond to topological depths. The AFM images show that the films are homogeneous for 32 × 32 μm BDD/P3MT at 500 mV (Figure 8). The thickness of these films was measured to be ~21.4 nm from the scale of the 3D view of the AFM image [65].

#### 6.1.3. Electrochemical Behaviour of GA on BDD and BDD/P3MT

**Figure 9:**
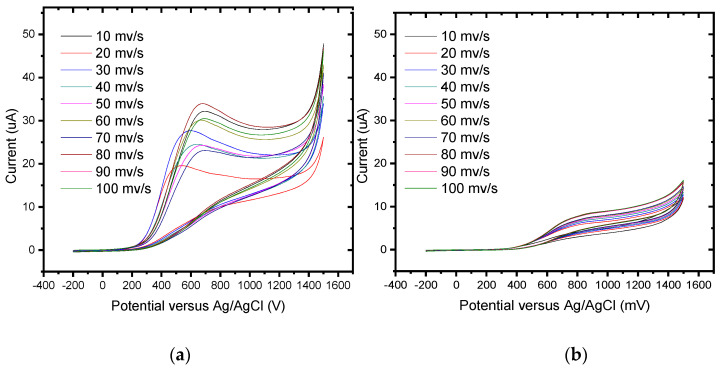
Cyclic voltammograms of 0.1 mM gallic acid in phosphate buffer solution (pH 6.6) at (**a**) the unmodified BDD electrode and (**b**) BDD/P3MT in the potential range from −200 mV to 1500 mV at scan rates of 10–100 mV/s.

**Figure 10:**
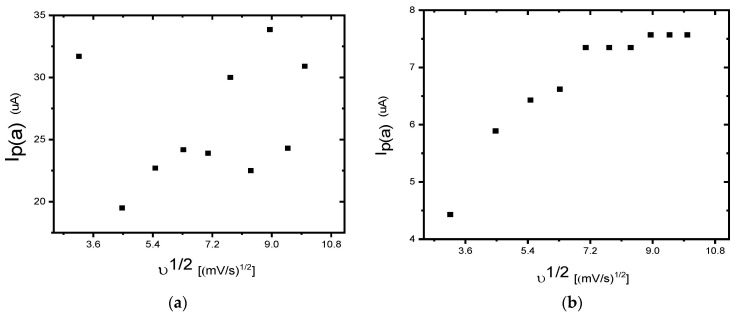
Calibration plot obtained for the scan rate study by cyclic voltammetry of 0.1 mM gallic acid in phosphate buffer solution (pH 6.6) at the (**a**) unmodified BDD electrode and (**b**) BDD/P3MT in the potential range from −200 mV to 1500 mV at scan rates of 10–100 mV/s.

The analytical behaviour of gallic acid (GA) was studied at the BDD unmodified electrode and BDD/P3MT chemical sensor in 0.1 M phosphate buffer at pH 6.6 at different scan rates from 10 to 100 mV/s (Figure 9 and Figure 10). An irreversible anodic CV wave was observed at 600 mV vs. Ag/AgCl in the electrochemical window of −200 mV to 1500 mV. The anodic current corresponds to the direct electrochemical oxidation of the GA to its quinone.

The BDD indicated an unstable, irregular, and unpredictable shift of the peak potential towards more positive potentials for the five repeated CV higher scan rates (Figure 10a). This irregular behaviour might be linked to the production of electro-inactive species that block the electrode surface, such as polygallic acid. The BDD/P3MT sensor showed that the anodic current corresponding to GA oxidation increased linearly with the square root of the scan rate (Figure 10b), indicating that oxidation is a diffusion-controlled process at the electrode surface. There was a positive linear shift of the oxidation peak potential E_p_ (for the wave observed) at increasing scan rates (10–100 mV/s). The height of the anodic peaks increased linearly with the gallic acid concentration until a steady state was reached. This behaviour has been observed in the anodic oxidation of other phenolic compounds, such as phenol, on BDD electrodes due to the deposition of organic films on the BDD surface that deactivate the electrode [42]. This deactivation is due to the formation of phenoxy radicals and subsequent oxidation to the corresponding phenoxy cation, which adsorbs onto the BDD electrode.

**Figure 11:**
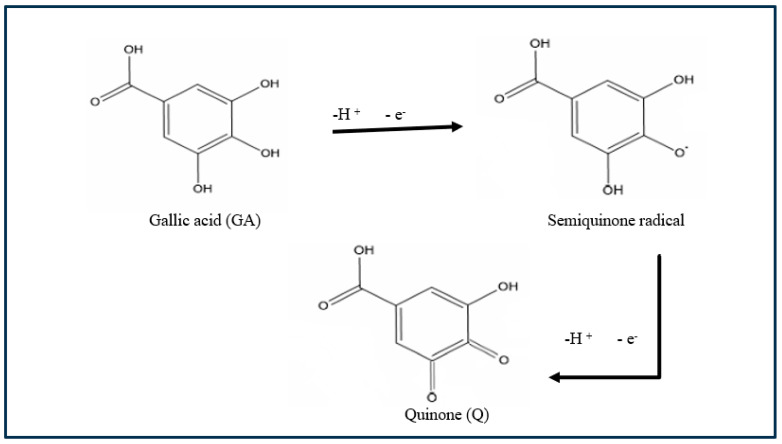
Electrochemical degradation of gallic acid.

GA is an electroactive molecule distinguished by particular redox reactions caused by the presence of three hydroxyl groups in the aromatic structure. The two GA hydroxyl groups are deprotonated, while two electrons and two protons are generated; the GA is irreversibly oxidised first into a semi-quinone radical and then into quinone, according to the equation (Figure 11). The semi-quinone is highly unstable and may rapidly decay through dimerization or polycondensation reactions. The above findings strongly suggest the exclusion of the possibility of dimerisation or polycondensation reactions.

The initial step involves the irreversible oxidation of GA to the semi-quinone radical cation (GA^+^) by the irreversible electron transfer process. Next, the formed radical cation loses a proton to form the semi-quinone radical (GA^−^). The one-electron oxidation product (semi-quinone radical, GA^−^) is followed by a second irreversible electron transfer to the quinone cation (GA^+^). Finally, deprotonation of the quinone cation (GA^+^) completes the overall two-electron process to give the quinone (GAO), which appears as an irreversible peak. Based on the previous results, the oxidation mechanism of gallic acid is described in Figure 11.

### 6.2. Analytical Response of GA

**Figure 12:**
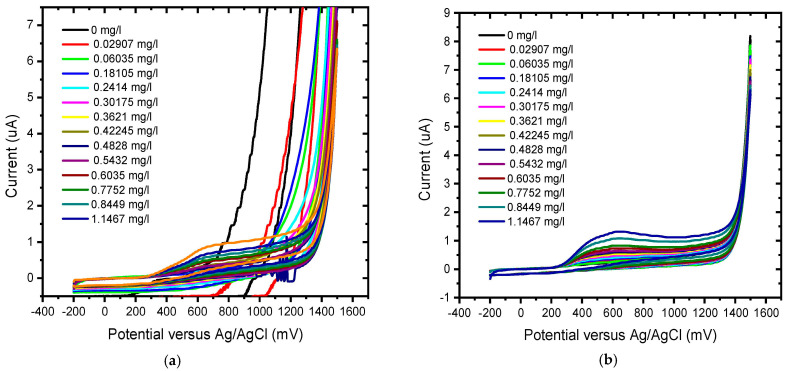
Concentration study of 0.1 mM gallic acid (0 mg/L to 1.1467 mg/L) in phosphate buffer solution (pH 6.6) at the (**a**) unmodified BDD electrode and (**b**) BDD/P3MT in the potential range from −200 mV to 1500 mV.

**Figure 13:**
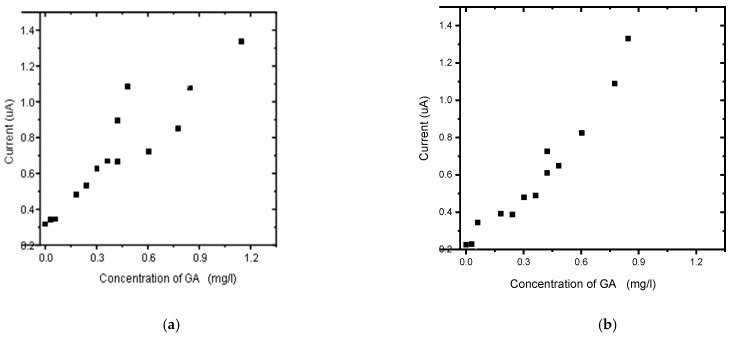
Calibration curves for the concentration study by CV of 0.1 mM gallic acid (0 mg/L to 1 mg/L) in phosphate buffer solution (pH 6.6) at the (**a**) unmodified BDD electrode and (**b**) BDD/P3MT in the potential range from −200 mV to 1500 mV.

CVs of the detection of GA on the BDD electrode and BDD/P3MT at pH 6.6 exhibited one irreversible anodic peak. BDD/P3MT exhibited a higher sensitivity towards the detection of GA than BDD, i.e., 1.23 × 10^−2^ mg/L versus 1.78 × 10^−2^ mg/L for BDD, both systems acquiring an R^2^ value of 0.98, as shown in Figure 12 and Figure 13.

**Figure 14:**
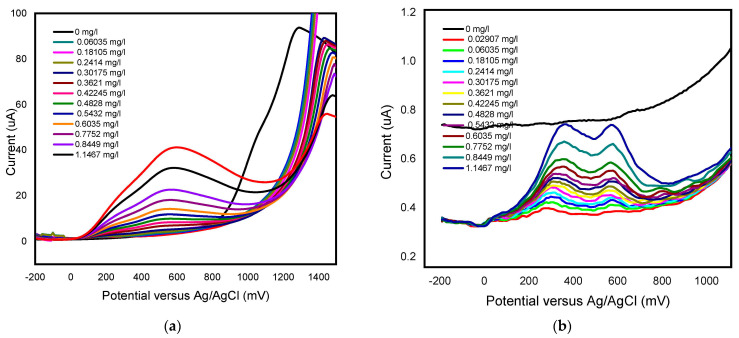
Concentration study of 0.1 mM gallic acid (0 mg/L to 1 mg/L) in phosphate buffer solution (pH 6.6) at the (**a**) unmodified BDD electrode and (**b**) BDD/P3MT in the potential range from −200 mV to 1500 mV at scan rate of 50 mV/s.

The responses of unmodified BDD electrode and BDD/P3MT sensor to 0.1 mM GA standard solutions were investigated by square wave voltammetry (SWV) in the applied potential window between −200 and +1500 mV at a scan rate of 50 mV/s for low and higher concentrations of GA, in Figure 12 and Figure 13, respectively. SWV is a more sensitive technique compared to CV. Figure 14 shows the SWV voltammograms for GA detection at low concentrations, in the range of 0 mg/L to 1 mg/L, exhibited one anodic peak at around 600 mV for both unmodified BDD and BDD/P3MT.

**Figure 15:**
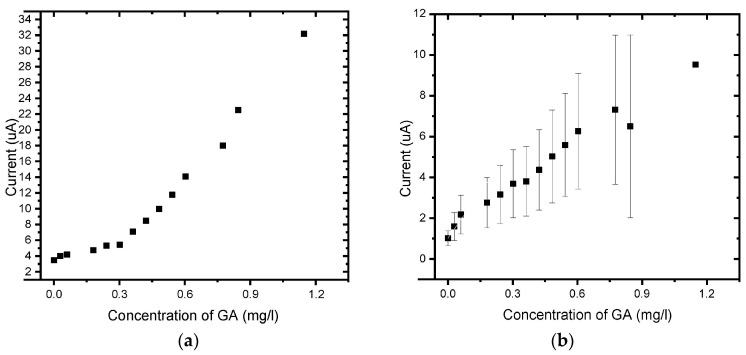
Calibration curve of the concentration study by SWV of 0.1 mM gallic acid (0 mg/L to 1 mg/L) in phosphate buffer solution (pH 6.6) at the (**a**) unmodified BDD electrode and (**b**) BDD/P3MT in the potential range from −200 mV to 1500 mV at a scan rate of 50 mV/s (n = 3).

The calibration curves in Figure 15, spanning a concentration range of 0–1 mg/L, reveal that BDD/P3MT displayed a slightly higher sensitivity (5.33 × 10^−5^ mg/L) towards the detection of GA than unmodified BDD (4.87 × 10^−5^ mg/L). BDD/P3MT also showed a higher R^2^ value of 0.99 compared to 0.94 for BDD. However, LOD and LOQ values obtained for BDD were 0.28 mg/L and 0.95 mg/L compared to the values for BDD/P3MT, i.e., 0.76 mg/L and 2.53 mg/L, respectively. The coefficient of determination (R^2^) of the calibration curve was used to evaluate the linearity of the curve. The limit of detection (LOD) and limit of quantitation (LOQ) were calculated as 3.3 σ/s and 10 σ/s, respectively, where σ is the standard deviation of the response, and S is the slope of the calibration curve.

**Figure 16:**
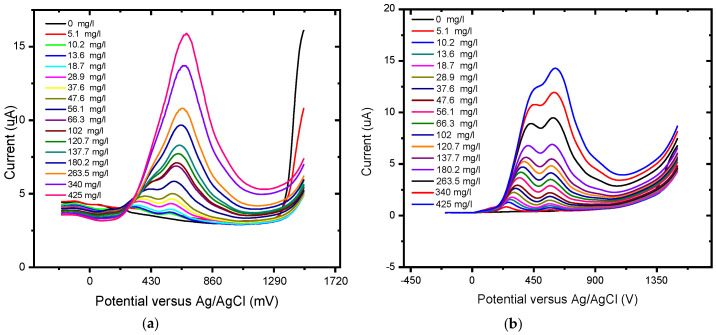
SWV concentration study of 0.1 mM gallic acid (0 to 440 mg/L) in phosphate buffer solution (pH 6.6) at the (**a**) unmodified BDD electrode and (**b**) BDD/P3MT in the potential range from −200 mV to 1500 mV at a scan rate of 50 mV/s (n = 3).

**Figure 17:**
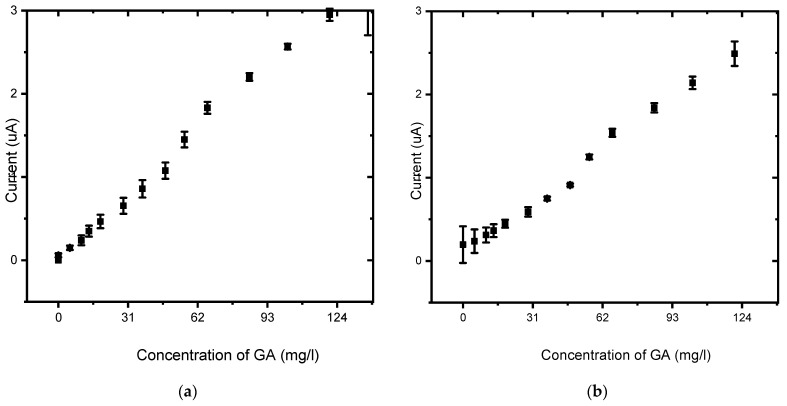
Calibration curve for 0.1 mM gallic acid response measured by SWV (0 mg/L to 124 mg/L) in phosphate buffer solution (pH 6.6) at the (**a**) unmodified BDD electrode and (**b**) BDD/P3MT in the potential range from −200 mV to 1500 mV at a scan rate of 50 mV/ (n = 3).

The responses of the unmodified BDD electrode and BDD/P3MT to 0.1 mM GA standard solutions for higher concentrations were investigated by square wave voltammetry (SWV) in the applied potential window between −200 and +1500 mV at scan rates of 50 mV/s (Figure 16) and 10 mV/s (Figure 17) versus Ag/AgCl. The voltammograms for BDD and BDD/P3MT at 50 mV/s depict the two anodic current peaks that are detectable at 400 mV/s and 600 mV versus Ag/AgCl. These peaks corresponded to the direct electrochemical oxidation of the gallic acid and were not observed in the CV study. However, the peak representing the semi-quinone formation was no longer present at higher concentrations (range 0 mg/L to 200 mg/L) for BDD electrodes. Meanwhile, for the BDD/P3MT system, this formation could also be seen at higher concentrations of GA. Both systems responded well to the detection of GA over a wide range. Unmodified BDD displayed a higher current response to GA compared to BDD/P3MT. In repeated studies (n = 3), the data obtained at BDD/P3MT electrodes produced larger error bars compared to unmodified BDD. However, BDD/P3MT depicted a more linear relationship for the response to the addition of gallic acid concentration in the electrochemical window. Competing mechanisms of adsorption and electrochemical oxidation complicate the analysis of GA electrochemical oxidation.

**Figure 18:**
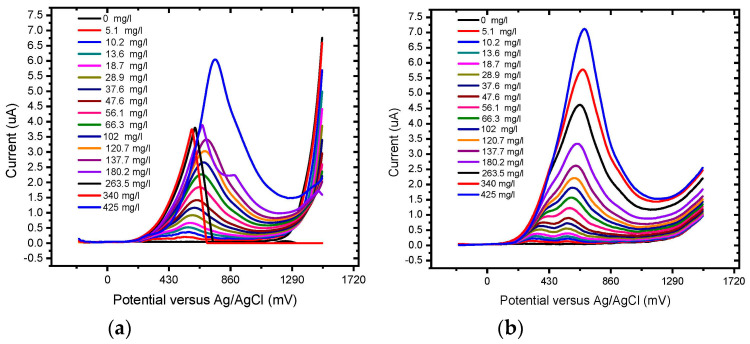
SWV concentration study of 0.1 mM gallic acid (0 mg/L to 200 mg/L) in phosphate buffer solution (pH 6.6) at (**a**) BDD and (**b**) BDD/P3MT in the potential range from −200 mV to 1500 mV at a scan rate of 10 mV/s.

**Figure 19:**
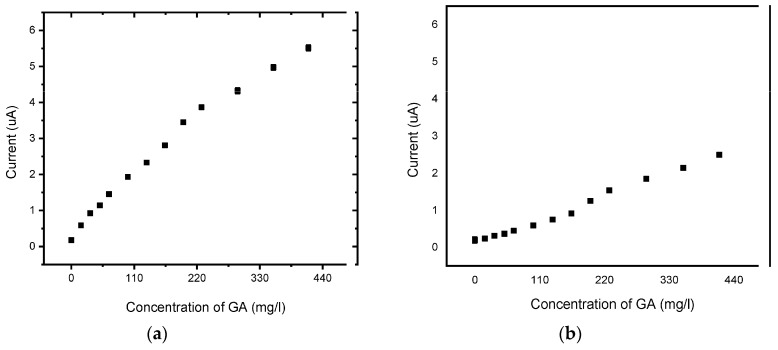
Calibration curve for the concentration response of 0.1 mM gallic acid measured via SWV (0 mg/L to 440 mg/L) in phosphate buffer solution (pH 6.6) at (**a**) BDD and (**b**) BDD/P3MT in the potential window of −200 mV to 1500 mV at a scan rate of 10 mV/s (n = 3).

The experiment was repeated for BDD and BDD/P3MT at a slower scan rate of 10 mV/s. The calibration curve showed improved linearity for the detection of GA and better reproducibility at the slower scan rate (Figure 18 and Figure 19) compared to BDD and BDD/P3MT at 50 mV/s. Furthermore, Table 2 depicts the sensitivity, LOD, and LOQ values for all systems in the same order of magnitude, with the system at 10 mV/s displaying a slight advantage in all three parameters.

Phenolic compounds, typically regarded as antioxidants, can also display pro-oxidant properties under certain environmental conditions. This kind of dual anti/pro-oxidant behaviour of these compounds is considered concentration-dependent and controlled by environmental factors such as the pH and the presence of transition metals. Previous studies suggest that the combination of acidic conditions and a low polyphenol concentration results in pro-oxidant activity [101,102,103,104].

The pro-oxidant behaviour has numerous implications in the context of oxidative stress and its related health risks. The data gathered so far strongly indicate that the role of polyphenols in the oxidative stress context is complex and needs to be fully understood to take advantage of their chemical behaviour for protecting human health [101].

The polyphenols’ in vivo structure and the antioxidant capacity relationship still need to be better understood. Still, the polyphenols’ antioxidant capacity in vitro is well established and closely related to their redox behaviour and electron transfer reactions. Moreover, although polyphenols are commonly known for their antioxidant effects that contribute positively to oxidative stress protection and balance, they can also exert a dangerous pro-oxidant action, producing free radicals under certain physiological conditions. However, this pro-oxidant activity may also be essential for the polyphenols’ ability to work as antimicrobial, antipathogenic, and therapeutic agents, and defence or deleterious agents, in apoptosis, in immunological defence mechanisms, and in ageing processes. Such duality depends on the physiological conditions and the polyphenols’ chemical properties.

Electrochemical investigations employing several hydroxycinnamic acids revealed that the phenolic’s electroactive components mostly determine their redox behaviours. Simultaneously, the double bond extension, methoxyl, and other non-electroactive groups are required for the electrochemical profile and antioxidant action. The C2=C3 double bond conjugation benefitted electron delocalisation, lowering the oxidation peak potential and increasing electrogenerated radical production in hydroxycinnamic acid derivatives [105].

Hydroxybenzoic acids can scavenge free radicals. Furthermore, these derivatives generally present a low redox potential and high electron donor ability. Since the radical scavenging of hydroxybenzoic acids is a dynamic process, the relatively small size of such a class of natural phenolic antioxidants improves their diffusion properties, which may positively influence their overall antioxidant capacity. In turn, non-electroactive electron donor substituents increase the electron spin density on the phenolic section, thus enhancing their reducing power. In addition, gallic acid possesses a catechol region [105].

Table 3 displays the comparison of analytical performance for GA between the proposed sensor and published results. We successfully developed a simple electrochemical platform for the fast, sensitive, and selective detection of GA The proposed BDD/P3MT chemical sensor has been shown to have good performance based on the electrochemical parameters such as the detection limit, linear range, and sensitivity.

### 6.3. Quantification of GA in Real Tea Samples

The quantification of gallic acid present in the different tea types was performed using two independent quantification methods. Method one was based on a regression equation obtained from a calibration curve for standard gallic acid oxidation at the BDD/P3MT electrode in PBS, pH = 6.6. The second method was the benchmark Folin –Ciocalteu method, which is based on the development of a chromophore of gallic acid using the Folin-C reagent for subsequent spectroscopic detection.

#### 6.3.1. Method 1—Screening by a Standard GA Curve

In a similar electrochemical experiment as described before for the characterization of the electrodes prepared in this work, we connected BDD/P3MT modified electrodes as the working electrode vs. the Ag/AgCl reference electrode. The oxidation of standard solutions of gallic acid was measured as a function of concentration in a PBS (pH = 6.6) electrolyte. All measurements were performed by SWV at 10 mV/s. The linear response of the peak current at 600 mV as a function of concentration was modelled as a linear regression line, with R^2^ = 0.94 (Figure 15). The concentration of total phenol compounds in all tea samples was determined as mg/L of gallic acid equivalents (GAEs). The TPC of tea samples was calculated using the gallic acid reference curve (*y* = 1.52 × 10^−5^*x* + 1.06 × 10^−6^; R = 0.98) established in a PBS medium.

Ten samples of rooibos tea, black tea, and green tea were screened for the presence of gallic acid. Rooibos tea produced 3/10 samples with a measurable phenol content, green tea produced 10/10 samples, and black tea produced 5/10 samples.

**Figure 20:**
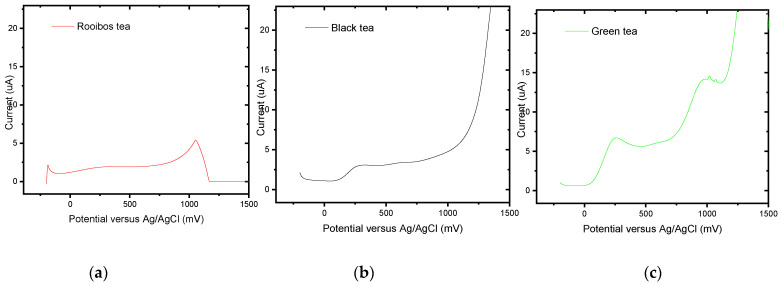
Examples of screening actual tea samples with a positive gallic acid indication via SWV for (**a**) rooibos, (**b**) black, and (**c**) green tea infusions at BDD/P3MT in the potential range from −200 mV to 1500 mV at a scan rate of 10 mV/s.

In recent years, research on polyphenols, a significant class of naturally occurring antioxidants found in various foods and beverages, has received considerable attention from both scientific and general audiences. One of the most significant component families in tea, the polyphenol family, is responsible for both the primary health benefits and the flavour and taste of tea. Total polyphenolic content (TPC) quantification is now a standard procedure for evaluating the quality of tea. Total polyphenols in tea samples significantly impact the quality of tea, affecting the biological properties. Literature reports indicate that, while any other oxidizable components present in the beverages will also contribute at some potentials, it is only the phenolics that are expected to produce a current at potentials less than 400 mV [25,48,61,62,63,64]. This can be seen by the first oxidation peak at 200–500 mV in tea profiles for black, green, and rooibos teas that produced acceptable voltammograms. The voltammograms were very similar to green and black teas (Figure 20). An initial peak was evident at about 200 mV, followed by a smaller peak at 500–700 mV. The black tea had only one peak at 200 mV. In the case of black tea, it is expected that most of the pyrogallol groups would have been oxidized by polyphenol oxidase during tea fermentation. The position of the cyclic voltammetry peaks reveals many phenolics with catechol and gallate groups (200 mV peak) in all the samples. The peak at 200 mV similarly indicates the level of phenolics in green teas. Still, it was less effective for black tea and rooibos tea due to electrode contamination from phenolic oxidation products produced in situ.

The total phenolic content of the extract is expressed as mg/L gallic acid equivalents (GAEs) per gram of sample at dry weight (mg/L/g). The total phenolic content of all samples was calculated according to the following formula:

GAEs (mg/L/g) = C × V/g, (1)

where C = the total phenolic content in mg GAEs/g dry extract, C = the concentration of gallic acid obtained from the calibration curve in mg/L/mL, V = the extract volume in ml and m = the mass extract in grams. The contents were expressed as gallic acid equivalents (mg/L GAEs/g).

The distributions of the TPC in rooibos tea (Table A1), green tea (Table A2), and black tea (Table A3) are shown as an Appendix A. The TPC for green tea samples was calculated to be 672.48 ± 48.23 GAEs/mg/L. The values for rooibos tea were observed to be the lowest of all values calculated, at 3.30 × 10^−4^ ± 2.42 × 10^−4^ GAEs/mg/L. The TPC for black tea was calculated to 594.44 ± 43.47 GAEs/mg/L (Table 4). Green tea had the highest TPC compared to rooibos tea and black tea. This is corroborated by literature records that reported that rooibos teas contain the lowest amount of polyphenols compared to green tea or black tea, and green teas contain the highest amount [25,94,115].

#### 6.3.2. Method 2—Determining the TPC by the Folin–Ciocalteu Method

**Figure 21:**
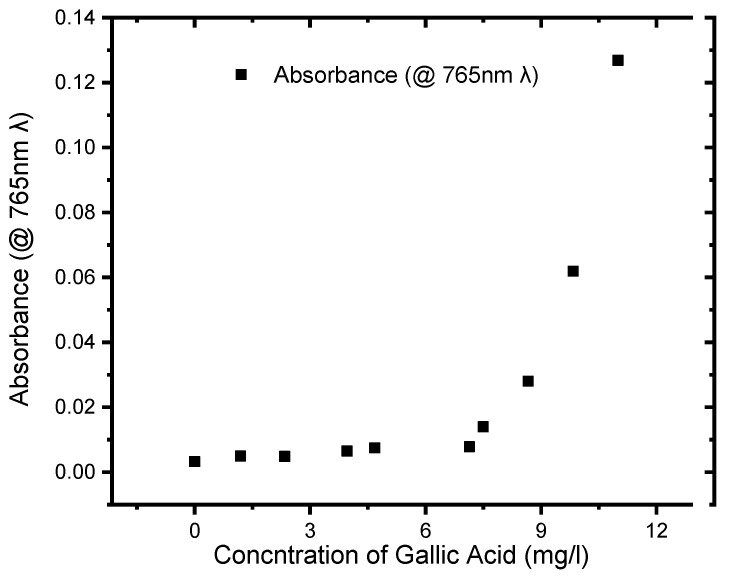
Calibration curve for gallic acid obtained by the Folin–Ciocalteu reaction method at 765 nm.

The Folin–Ciocalteu method is an optical method that uses a special reagent (Folin-C reagent) to produce a chromophore that can be related quantitatively to the total phenolic content of food samples. The procedure consists of sonicated water extraction followed by a reaction with the Folin-C reagent. At 765 nm, the colourimetric reaction is measured and compared to a standard curve prepared using gallic acid standard solutions. The total phenolic content of the tea extracts in our study was determined using a spectrometric approach based on the Folin–Ciocalteu reagent, as reported in (Adedapo et al., 2008). The Folin–Ciocalteu reagent decreases polyphenol-containing tea samples, resulting in a blue-coloured complex. A gallic acid calibration curve was used to determine the phenolic content of instant teas. Aliquots of 40 μL, 0–11 mg/L gallic acid solutions were combined with 0.2 mL (ten-fold diluted) of Folin–Ciocalteu reagent and 0.6 mL of (75%) sodium carbonate. The tubes were vortexed for 10 s before being allowed to incubate for 2 h at 25 degrees Celsius. After 2 h of incubation at 25 °C, absorbance at 765 nm was measured against a reagent blank using a ThermoFisher GENESYSTM 30 Visible Spectrophotometer UV-Vis Spectrophotometer. Based on the calibration curve, the total phenolic content was calculated as mg/L gallic acid equivalents using the following equation: y = 0.0011x + 0.00296, R^2^ = 0.88, where x was the absorbance and y was the gallic acid equivalent (Figure 21). As stated before, a similar process was used for the extract in the construction of the calibration curve. The total phenolic content was expressed as mg/L of gallic acid equivalents (GAEs) per g of tea. The responses for the GA in associated concentration ranges were linear based on a 1/x weighted linear regression analysis (Figure 21).

The Beer–Lambert rule states that at a given concentration of a sample, the absorbance is proportional to the concentration. Linearity was maintained in the concentration range of 0.40 to 11.00 mg/L (R^2^ = 0.88). LOQ was calculated as 3.3 σ/s, where σ is the standard deviation of the response, and S is the slope of the calibration curve and was found to be 10371 mg/L. The LOD was calculated as 3.3 σ/s, where σ is the standard deviation of the response, and S is the slope of the calibration curve and was calculated to be a value of 33.92 mg/L.

The values for the TPC obtained for each sample using the proposed sensor and the Folin–Ciocalteu (FC) method are shown in Table 5, together with the relative error between the results obtained applying the two methods. Rooibos tea samples are seen to have a lower absorbance than black and green tea for all absorbance bands; this is a result of the fact that rooibos tea has a lower polyphenol content than green tea and black tea. The order of the TPC of tea polyphenols can be seen to be green > black > rooibos teas.

The results obtained from the analysis were more significant than those obtained using the BDD/P3MT sensor. As previously stated, the polymer-modified surface allows for ease of reusability; additionally, when combined with square-wave voltammetry, it allows low and high detection and quantification limits. The results support the conclusion that the proposed BDD/P3MT sensor, in conjunction with square-wave voltammetry, is a promising methodology for determining the TPC in tea samples without the need for elaborate sample preparation and pre-concentration steps, significantly reducing the analysis.

This characteristic is related to the distinct oxidizing agents utilized in each case, namely, the chemical reagents used in the FC technique and the voltage supplied to the electrode surface for the oxidation process in the electrochemical sensors. The FC reagents oxidize non-phenolic species present in the tea sample, but do not oxidize the phenolic compounds themselves. Additionally, the applied voltage (200 mV) solely oxidizes the phenolic chemicals. Despite this discrepancy, the accuracy offered by the BDD/P3MT sensor is equivalent to that of the FC technique, where the maximum relative error between the determinations was an average of 7%. The precision of the readings acquired using the two processes was comparable, showing the BDD/P3MT sensor to be a reliable and viable alternative for the determination of phenolic compounds in tea samples.

## 7. Conclusions

Polyphenols are the main components of tea, which are responsible for the antioxidant activities of tea beverages. The extraction, separation, identification, and analysis of individual polyphenols remain problematic due to their chemical complexity and common occurrence in plants. The present study was undertaken to assess the total polyphenols in South African tea types quantified as gallic acid equivalents.

Gallic acid was employed as a marker to represent the TPC because it is the indicator used in the Folin–Ciocalteu (FC) technique, which is the most frequently utilized method for TPC determination. The BDD/P3MT chemical sensor has demonstrated good analytical performance towards the detection of GA, such as the detection limit, limit of quantification, and sensitivity. The calibration plot is linear across the wide concentration range of 0 mg/L to 200 mg/L.

The BDD/P3MT sensor reported a TPC in green tea samples of 672.48 ± 48.23 GAEs/mg/L. The results for rooibos tea were found to be the lowest of those calculated, at 3.30 × 10^−4^ ± 2.42 × 10^−4^ GAEs/mg/L. The TPC for black tea was determined to be 594.44 ± 43.47 GAEs/mg/L. green tea exhibited the highest TPCs compared to rooibos and black teas.

The results obtained by both the BDD/P3MT sensor and by the FC method for the same samples showed that rooibos tea contains the lowest polyphenol content compared to green tea or black tea, with green tea showing the highest amount.

The FC reagents oxidize any non-phenolic species present in the tea sample rather than the phenolic compounds.

Although phenols easily foul the BDD transducer; the polymer-modified surface facilitates its reusability, as conductive polymers have been shown to offer antifouling characteristics. Moreover, it provides good detection and quantification limits when paired with square-wave voltammetry. The findings support the conclusion that the proposed BDD/P3MT sensor, when combined with square-wave voltammetry, is a viable technology for measuring the TPC in tea samples. This eliminates the need for complicated sample preparation and pre-concentration stages, considerably shortening the analysis.

These aspects contribute to BDD/P3MT’s benefits as a chemical sensor that allows quick one-step TPC detection. Electrochemical detection has been shown to be a convenient, economical, sensitive, selective, and quick method for detecting the total tea polyphenol concentration.

Providing the total polyphenol content and quantification in this way can fulfil the data for the tea industry’s quality control requirement. Direct electrochemical detection of tea constituents as a single voltametric signal is highly recommended in this respect. The food and food safety industries could benefit from this high-throughput method’s quick polyphenol determination.

The authors state that they do not have any known competing financial interests or personal ties that may have influenced the work disclosed in this study.

## Figures and Tables

**Table 1:** Roughness assessment of unmodified BDD and P3MT/BDD film by AFM.

Electrode	Image Size (µm)	Voltage of Cantilever	Surface Area (µm)
Unmodified BDD	8 × 8	500 mV	11.82 × 10^−3^
Unmodified BDD	8 × 8	1000 mV	37.76 × 10^−3^
Unmodified BDD	32 × 32	500 mV	0.526
P3MT/BDD	8 × 8	500 mV	5.40
P3MT/BDD	8 × 8	1000 mV	5.45
P3MT/BDD	32 × 32	500 mV	14.93

**Table 2:** Analytical parameters obtained via SWV and CV for the gallic acid electrochemical analysis.

	**Cyclic Voltammetry**					
	**Epa1**		**R^2^**	**Sensitivity** **(mg/L)**	**LOD** **(mg/L)**	**LOQ** **(mg/L)**
BDD	850 mV		0.94	4.87 × 10^−5^	0.50	1.62
BDD/P3MT	850 mV		0.99	5.33 × 10^−5^	1.30	4.30
	**Square Wave Voltammetry**					
	**Epa1**	**Epa2**	**R^2^**	**Sensitivity (mg/L)**	**LOD** **(mg/L)**	**LOQ** **(mg/L)**
BDD (low concentration—0 mg/L to 1 mg/L)		600 mV	0.94	1.52 × 10^−5^	0.13 mg/L	3.10 × 10^4^
BDD/P3MT (low concentration—0 mg/L to 1 mg/L)	400 mV	600 mV	0.98	4.28 × 10^−5^	0.39 mg/L	2.56 × 10^5^
BDD (high concentration— 0 mg/L to 420 mg/L)		600 mV	0.99	4.2 × 10^−8^	12	3.47 × 10^−2^
BDD/P3MT (high concentration—0 mg/L to 420 mg/L)	400 mV	600 mV	0.99	2.95 × 10^−6^	11	2.96 × 10^−2^

**Table 3:** Comparison of analytical performance for GA between the proposed sensor and published results.

Electrode	Method	Sample	Characteristics	Reference
A functionalized graphene oxide/poly (p-amino-hippuric acid)-sodium dodecyl sulfate nanocomposite modified glassy carbon electrode (APTS@GO/PPAH-SDS/GCE)	CV (cyclic voltammetry), DPV (differential pulse voltammetry), amperometry	Black tea Tap water	Linear range of 1.0 × 10^−3^ mg/L; LOD of 2.89 × 10^−3^ mg/L for GA (S/N = 3) using the amperometric method; determination in the real samples	[106]
Glassy carbon electrode modified with a Zn/Al-layered double hydroxide film	DPV	PBS, pH 3	Linear range of 0.68–102 mg/L; LOD of 2.72 mg/L for GA	[107]
Carbon paste electrode modified with multi-walled carbon nanotubes	DPV	PBS, pH 1.7	Linear range of 0.136–17.01 mg/L; LOD of 0.042 mg/L for GA	[108]
Carbon paste electrode modified with multi-walled carbon nanotubes containing iron oxides	DPV	PBS, pH 2	Linear range of 0.17–106.325 mg/L; LOD of 1747.13 mg/L for GA	[109]
Glassy carbon electrode modified with polyepinephrine	DPV	PBS, pH 2.5	Linear range of 0.085–25.51 mg/L LOD of 0.051 mg/L for GA	[110]
Graphite electrode modified with a dinuclear copper (II) octaazamacrocyclic complex in PVC matrix	SWV	PBS, pH 1.88	Linear range of 0.17–3.40 mg/L LOD of 0.11 mg/L for GA	[111]
Graphite electrode modified with thionine and nickel hexacyanoferrate	DPV	PBS, pH 2	Linear range of 0.04–0.17 mg/L LOD of 0.025 mg/L for GA	[112]
Glassy carbon electrode modified with cerium dioxide nanoparticles in a 0.02 M BrijA(R)35	DPV	pH 7.4; spices	Analytical range of 8.506–243 mg/L LOD of 2.0 mg/L	[113]
Activated screen-printed carbon electrode (ASPCE)	Amperometry	Green tea Apple juice	Linear range of 1.7 × 10^−3^–306.17 mg/L LOD of 5.27 × 10^−3^ mg/L	[114]
BDD/3PMT	Square wave voltammetry	PBS, pH 6.6	Linear range of 3–71 mg/L LOD of 11 mg/L LOQ of 2.96 × 10^−2^ mg/L	This study

**Table 4:** Total polyphenol contents of rooibos, black and green teas; the result are expressed in terms of GAEs/mg/L of the sample for three independent measurements (triplicate; n = 3).

Tea Type	Average Peak Current, uA (n = 3)	Average Concentration Method 1, mg/L (n = 3)	Average GAEs, mg/L (n = 3)
Rooibos tea	1.49 × 10^−6^ ± 3.14 × 10^−7^	1.09 ± 0.16	3.30 × 10^−4^ ± 2.42 × 10^−4^
Black tea	4.07 × 10^−6^ ± 6.46 × 10^−7^	17.01 ± 1.27	594.44 ± 43.47
Green tea	2.49 × 10^−6^ ± 1.11 × 10^−6^	17.34 ± 1.24	672.48 ± 48.23

**Table 5:** The total polyphenol content of one sample of rooibos tea, black tea, and green tea. The result obtained for the FC method compared to electrochemical method 2 was expressed in terms of GAEs/mg/L of the tea sample.

FC Method Results				
Tea Sample Name	Volume of Tea Sample Used (μL)	GAE Concentration by the FC Method (mg/L)	GAE Concentration Electrochemically Detected at the BDD/P3MT (Method 1) (mg/L)	The Error Associated with Method 2 Compared to the FC Method (%)
Rooibos Tea	2	21.34	0.06	0.26
	4	27.09	0.28	1.04
	6	35.30	1.10	3.12
	8	65.84	2.07	3.14
	10	78.10	4.23	5.42
	12	108.91	10.58	9.71
Black Tea	2	34.91	0.05	0.13
	4	82.55	0.24	0.28
	6	90.90	14.35	15.79
	8	168.79	1.07	0.64
	10	180.16	7.18	3.99
	12	239.91	17.95	7.48
Green Tea	2	46.39	3.16	6.81
	4	65.20	15.79	24.22
	6	88.62	10.96	12.36
	8	221.97	14.46	6.51
	10	205.00	22.04	10.75
	12	305.04	55.09	18.06

## Data Availability

The original contributions presented in the study are included in the article, further inquiries can be directed to the corresponding author.

## Appendix A

**Table A1:** Total polyphenol contents of rooibos tea, the results were expressed in terms of GAEs/mg/L of the sample for three independent measurements (triplicate; n = 3).

Tea Bag Sample Number	Average Rooibos Tea Peak Current, uA (n = 3)	Concentration Method 1, mg/L (n = 3)	GAEs, mg/L (n = 3)
1	9.99 × 10^−7^ ± 6.39 × 10^−7^	3.03 × 10^−2^ ± 0.62	4.17 × 10^−4^ ± 1.56
2	1.45 × 10^−6^ ± 9.37 × 10^−7^	8.49 × 10^−2^ ± 0.92	5.63 × 10^−7^ ± 3.83
3	1.76 × 10^−6^ ± 5.57 × 10^−7^	4.08 × 10^−2^ ± 0.54	5.73 × 10^−4^ ± 3.06
4	N/A	N/A	N/A
5	N/A	N/A	N/A
6	N/A	N/A	N/A
7	N/A	N/A	N/A
8	N/A	N/A	N/A
9	N/A	N/A	N/A
10	N/A	N/A	N/A
Average	1.49 × 10^−6^	1.09	3.30 × 10^−4^
STD	3.14 × 10^−7^	0.16	2.42 × 10^−4^

**Table A2:** Total polyphenol contents of black tea, the results were expressed in terms of GAEs/mg/L of the sample for three independent measurements (triplicate; n = 3).

Tea Bag Sample Number	Average Black Tea Peak Current, uA (n = 3)	Concentration Method 1, mg/L (n = 3)	GAEs, mg/L (n = 3)
1	3.79 × 10^−6^ ± 1.08 × 10^−6^	18.62 ± 1.06	616 ± 27.13
2	4.82 × 10^−6^ ± 1.62 × 10^−6^	19.77 ± 1.59	660 ± 32.62
3	2.31 × 10^−6^ ± 2.49 × 10^−6^	18.29 ± 2.44	594.86 ± 0.00
4	2.31 × 10^−7^ ± 2.77 × 10^−7^	17.22 ± 0.27	569.96 ± 19.58
5	2.36 × 10^−6^ ± 1.08 × 10^−6^	16.01± 1.06	530.80 ± 0.00
6	N/A	N/A	N/A
7	N/A	N/A	N/A
8	N/A	N/A	N/A
9	N/A	N/A	N/A
10	N/A	N/A	N/A
Average	4.07 × 10^−6^	17.01	594.44
STD	6.46 × 10^−7^	1.27	43.47

**Table A3:** Total polyphenol contents of green tea, the results were expressed in terms of GAEs/mg/L of sample for three independent measurements (triplicate; n = 3).

Tea Bag Sample Number	Average Green Tea Peak Current, uA (n = 3)	Concentration Method 1, mg/L (n = 3)	GAEs, mg/L (n = 3)
1	3.77 × 10^−6^ ± 4.34 × 10^−6^	18.60 ± 4.25	6.92 × 10^2^ ± 32.05
2	1.81 × 10^−7^ ± 4.81 × 10^−6^	16.61 ± 0.47	6.69 × 10^2^ ± 32.15
3	3.71 × 10^−6^ ± 1.83 × 10^−6^	18.54 ± 1.79	7.63 × 10^2^ ± 0
4	2.40 × 10^−6^ ± 1.13 × 10^−6^	17.25 ± 1.11	6.39 × 10^2^ ± 34.68
5	5.04 × 10^−6^ ± 1.64 × 10^−6^	19.84 ± 1.61	7.18 × 10^2^ ± 51.12
6	1.10 × 10^−6^ ± 1.79 × 10^−7^	15.98 ± 0.17	6.50 × 10^2^ ± 23.82
7	1.02 × 10^−6^ ± 9.26 × 10^−7^	15.91 ± 0.91	5.93 × 10^2^ ± 43.00
8	1.22 × 10^−6^ ± 9.02 × 10^−7^	16.10 ± 0.88	6.15 × 10^2^ ± 32.14
9	2.07 × 10^−6^ ± 8.69 × 10^−7^	16.94 ± 0.85	6.93 × 10^2^ ± 12.70
10	2.082 × 10^−6^ ± 8.54 × 10^−7^	17.67± 0.83	6.62 × 10^2^ ± 291.90
Average	2.49 × 10^−6^	17.34	672.48
STD	1.11 × 10^−6^	1.24	48.23
